# SHMP-Amended Ca-Bentonite/Sand Backfill Barrier for Containment of Lead Contamination in Groundwater

**DOI:** 10.3390/ijerph17010370

**Published:** 2020-01-06

**Authors:** Yu-Ling Yang, Krishna R. Reddy, Wen-Jie Zhang, Ri-Dong Fan, Yan-Jun Du

**Affiliations:** 1 Jiangsu Key Laboratory of Urban Underground Engineering & Environmental Safety; Institute of Geotechnical Engineering, Southeast University, Nanjing 210096, China; ylyang@seu.edu.cn; 2 Department of Civil & Materials Engineering, University of Illinois at Chicago, Chicago, IL 60607, USA; kreddy@uic.edu; 3 Department of Civil Engineering, Shanghai University, Shanghai 200072, China; zhwjlyl@163.com; 4 School of Materials Science and Engineering, Southeast University, Nanjing 210096, China; 101101786@seu.edu.cn

**Keywords:** soil-bentonite slurry walls, hydraulic conductivity, sorption, lead contamination, groundwater

## Abstract

This study investigated the feasibility of using sodium hexametaphosphate (SHMP)- amended calcium (Ca) bentonite in backfills for slurry trench cutoff walls for the containment of lead (Pb) contamination in groundwater. Backfills composed of 80 wt% sand and 20 wt% either Ca-bentonite or SHMP-amended Ca-bentonite were tested for hydraulic conductivity and sorption properties by conducting laboratory flexible-wall hydraulic conductivity tests and batch isothermal sorption experiments, respectively. The results showed that the SHMP amendment causes a one order of magnitude decrease in hydraulic conductivity of the backfill using tap water (1.9 to 3.0 × 10^−10^ m/s). Testing using 1000 mg/L Pb solution resulted insignificant variation in hydraulic conductivity of the amended backfill. Moreover, SHMP-amendment induced favorable conditions for increased sorption capacity of the backfill, with 1.5 times higher retardation factor relative to the unamended backfill. The Pb transport modeling through an hypothetical 1-m-thick slurry wall composed of amended backfill revealed 12 to 24 times of longer breakthrough time for Pb migration as compared to results obtained for the same thickness slurry wall with unamended backfill, which is attributed to decrease in seepage velocity combined with increase in retardation factor of the backfill with SHMP amendment. Overall, SHMP is shown to be a promising Ca-bentontie modifier for use in backfill for slurry trench cutoff wall for effective containment of Pb-contaminated groundwater.

## 1. Introduction

Soil-bentonite (SB) slurry trench cutoff walls usually consist of backfill composed of sodium (Na) bentonite mixed with either in-situ excavated soil or appropriate imported soil, and are commonly used in the U.S. as vertical barriers to prevent lateral migration of contaminated groundwater [1]. The SB slurry walls may be used as an interim measure for the short-term containment of impacted groundwater until an efficient and effective treatment technology is developed and deployed [2]. Low permeability and the contaminant sorption capacity are the two dominated properties of the backfill that control the containment performance of the walls [3,4]. To enhance service life and/or service performance of the SB slurry walls, novel bentonites including multiswellable bentonite MSB, contaminant-resistant bentonite SW101 [5], and bentonite-polymer composite BPC [6] were developed to reduce hydraulic conductivity of the SB backfills. In addition, sorbing materials such as zeolites [7,8], activated carbon [9], and natural humus [10] are adopted as amendment to enhance contaminant sorption capacity of the SB backfills.

For countries such as China and India, where high quality natural Na-bentonite is scarce and groundwater contamination containment is imminently needed, relatively abundant calcium (Ca) bentonite could be considered as an alternative material for use [7,11,12,13]. However, proper amendment is required for the Ca-bentonite before its use because its insufficient swelling and sorption capacities relative to Na-bentonite may result in high hydraulic conductivity and low contaminant sorption in the sand/Ca-bentonite backfills.

Among various amendments, a non-toxic phosphate dispersant, specifically sodium hexametaphosphate (SHMP), has been demonstrated to provide positive effects on: (1) enhancing heavy metal sorption capacity of the kaolin clay [14]; (2) improving dispersivity of the kaolin in saline water [15], and (3) decreasing hydraulic conductivity of the natural clayey soils when using tap water as permeated liquid [16]. It is also shown by the authors in previous studies that 2 wt% SHMP (dry weight ratio of SHMP to bentonite) can significantly improve workability of Ca-bentonite/water slurry and reduce seepage through the sand/Ca-bentonite backfills [12,17,18]. However, the containment performance of the backfill comprising SHMP-amended Ca-bentonite has not been systematically investigated.

This paper presents an experimental investigation to quantify the contaminant containment performance of sand/SHMP-amended Ca-bentonite backfill by resorting to flexible-wall hydraulic conductivity tests, batch isothermal sorption tests, and one-dimensional migration modeling. The unamended backfill containing unamended Ca-bentonite was also tested for comparison purposes. Lead (Pb) contamination is chosen in this study as it is commonly found at contaminated soil/groundwater sites in China [19,20,21,22].

## 2. Materials and Methods

### 2.1. Materials

A clean sand known as Three River Sand, a powdered Ca-bentonite (CaB), and granular SHMP were used in this study. The CaB was supplied by the Colloid Environmental Technologies Company (Hoffman Estates, IL, USA), and the sand was obtained from a quarry located in Beloit, (WI, USA). The SHMP was purchased from Humboldt Manufacturing Co. (Elgin, IL, USA). In addition, a powdered SHMP-amended Ca-bentonite (SHMP-CaB) was prepared by combining the CaB and SHMP. In brief, preparation of the SHMP-CaB included creating slurry with SHMP-to-CaB ratio of 2% and water-to-solid ratio of 1:2, curing the slurry at room temperature for 24 h, and oven drying for another 24 h, grinding the dried soil, and passing through No. 200 standard sieve. Details of this SHMP-CaB preparation procedure were provided in Yang et al. [18,23].

### 2.2. Hydraulic Conductivity Tests

**Figure 1:**
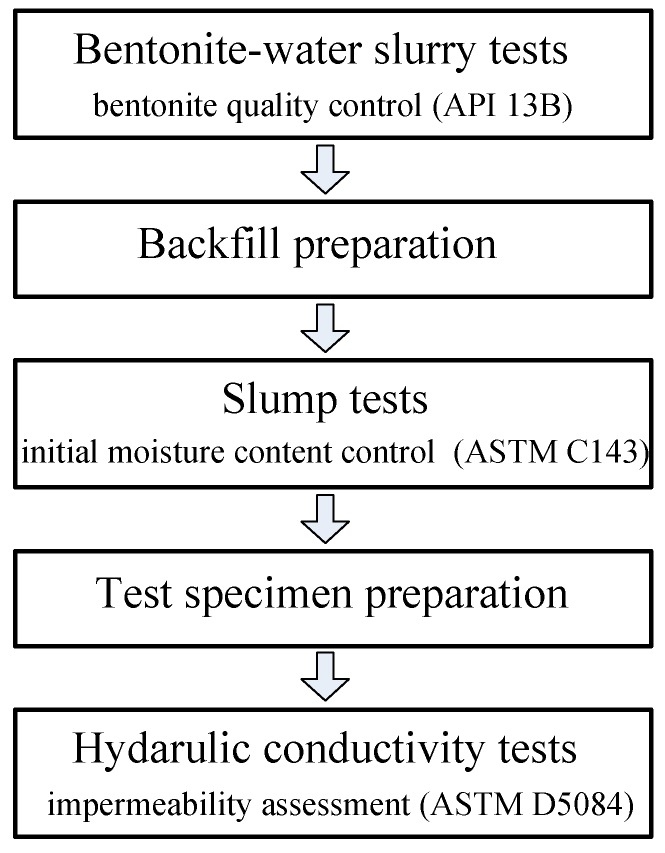
Scheme for flexible-wall hydraulic conductivity tests.

Flexible-wall hydraulic conductivity tests were conducted on two backfills: (1) unamended backfill (i.e., backfill without SHMP amendment) denoted by 20CaB, and (2) SHMP amended backfill, denoted by SHMP-20CaB. The backfills were composed of 80 wt% sand and 20 wt% either CaB or SHMP-CaB as recommended by Yang et al. [17]. The specimen preparation and the test scheme are shown in Figure 1. The tap water-CaB slurry, although CaB content reached 30 wt%, was found to exhibit insufficient Marsh viscosity (e.g., <35 s) and excess filtrate loss (e.g., >25 mL) after 24 h hydration, hence considered not suitable for backfill preparation. Therefore, tap water was used in this study to adjust moisture content of the 20CaB backfill until a target slump height [24] of 125 mm, as recommended by Evans [25], was achieved. For the SHMP-20CaB preparation, tap water-SHMP-CaB slurry composed of 20 wt% amended bentonite was prepared and used to increase moisture content of the backfill to satisfy the target slump height. The SHMP-CaB slurry had Marsh viscosity of 39 s, filtrate loss of 22.6 mL, density of 1.15 g/cm^3^, and pH of 6.8 after hydration for 24 h. To control bentonite content in the backfill to be constant 20 wt%, additional amount of sand was added with the slurry during the slump tests. Moisture content of the prepared 20CaB backfill and SHMP-20CaB backfill at the target slump was 29.6% and 30.0%, respectively.

Hydraulic conductivity tests were performed in accordance with the falling headwater-rising tailwater method (method C) in accordance with ASTM D5084 [26]. Because the backfill was very soft under high slump value, the hydraulic conductivity specimens were prepared, assembled, and back-pressure saturated in a rigid acrylic cylinder as described by Malusis et al. [27]. Average effective stress of 34.5 kPa and hydraulic gradient less than 30 were applied during the permeation process. Tap water was permeated through each specimen to determine the baseline hydraulic conductivity (*k*_w_), until the following termination criteria specified in ASTM D5084 were achieved: at least four hydraulic conductivity values were obtained over a time interval in which (1) ratio of outflow-to-inflow rate was between 0.75 and 1.25, and (2) the hydraulic conductivity was steady with four continuous hydraulic conductivities within ±25% of their mean value and also insignificant upward or downward trend was observed for the hydraulic conductivity with test duration. Next, the testing was continued by substituting the tap water with 1000 mg/L Pb solution until the end of the testing. The hydraulic conductivity with Pb contaminated solution (*k*_c_) was then calculated, and the effluent volume as well as Pb concentration in the outflow liquid was monitored as the tests progressed. After the testing, the specimens were disassembled, and final dimensions and moisture content of each specimen were determined. All hydraulic conductivity tests were conducted in duplicate to ensure repeatability of the test results.

### 2.3. Batch Sorption Tests

First, a stock solution with Pb concentration of 40,000 mg/L was prepared by dissolving predetermined amount of lead nitrate, Pb(NO_3_)_2_, in deionized water. Then, the stock solution was diluted using deionized water to yield a series of Pb solution with initial concentration (*C*_0_) of 5 to 40,000 mg/L. The pH and electrical conductivity (*EC*) of each Pb solution was measured as per ASTM E70 [28] and ASTM D1125 [29], respectively.

Both unamended backfill (20CaB) and SHMP-amended backfill (SHMP-20CaB) were used for batch sorption tests. The sorption tests were conducted according to the procedure described in ASTM D4646 [30] except the solid-to-liquid ratio of 1:10 was used as recommended by Reddy et al. [31]. The backfill samples used comprised of 8 g of sand and 2 g of either CaB or SHMP-CaB. Each backfill sample was placed in a 200 mL plastic bottle, and then 100 mL of selected Pb solution (with known concentration) was added to it. The bottle was then mixed end-over-end at 29 rpm in a mechanical tumbler for 24 h at room temperature of 20 °C. Then, the supernatant was collected by centrifugation and filtration (using 0.45 μm filter paper). The concentration of Pb in the supernatant which represents the equilibrium concentration (*C*_e_) was measured using an atomic absorption spectrophotometer as per the USEPA standard procedure 7000B [32].

The amount of Pb sorbed onto unit dry mass of backfills (*q*_e_) was calculated using following equation:

(1) $$q_{e}=\frac{(C_{0}-C_{e})M_{s}}{V_{L}}$$

where *q*_e_ is with unit of mg/kg, *M*_s_ is the mass of dry backfill (0.01 kg in this study), *V* is the volume of added Pb solution (0.1 L in this study), *C*_0_ and *C*_e_ are initial Pb concentration and equilibrium Pb concentration in the solution, respectively (both with unit of mg/L).

Two sorption isotherm models, Langmuir model and Freundlich model, were applied to investigate the *q*_e_-*C*_e_ relationship. The Langmuir model is given by below equation [33]:

(2) $$q_{e}=\frac{K_{L}q_{m,L}C_{e}}{1+K_{L}C_{e}}$$

where *q*_m,L_ is the sorption capacity (unit of mg/kg); *K*_L_ is the Langmuir isotherm constant (unit of L/mg). The Freundlich model is given as follows [34]:

(3) $$q_{e}=K_{F}C_{e}^{1/n_{F}}$$

where the *K*_F_ is the Freundlich constant (unit of L/kg); *n*_F_ is the sorption intensity (dimensionless). An *n*_F_ value between 1 and 10 is associated with favorable sorption.

The partition coefficient *K*_p_ was calculated using the Freundlich regressed model for Pb equilibrium concentration *C*_e_ of 1000 mg/L. Its secant line definition as suggested by Shackelford [35] was adopted for this calculation.

### 2.4. Solute Transport Equation

Assuming an one-dimensional condition, constant linear average seepage velocity, isothermal linear sorption, and no chemical/biological reactions, the governing equation for contaminant transport through a homogeneous and isotropic strata with infinite width can be described by the following initial condition, boundary conditions, and advection-dispersion-reaction equation [1]:

Initial condition:

*c*(*x*,0) = 0, *x* > 0 (4)

Boundary conditions:

(5) $$\begin{array}{l} c\left(0,t\right)=c_{0}{,}^{}t\geq 0\\ c\left(\infty ,t\right)=0{,}^{}t\geq 0 \end{array}\}$$

Advection-dispersion-reaction equation:

(6) $$c\left(x,t\right)=\frac{c_{0}}{2}\left[\mathrm{erfc}\left(\frac{R_{d}x-vt}{2\sqrt{DtR_{d}}}\right)+\exp\left(\frac{vx}{D}\right)\mathrm{erfc}\left(\frac{R_{d}x+vt}{2\sqrt{DtR_{d}}}\right)\right]$$

where *c*_0_ and *c* are initial contaminant concentration and contaminant concentration at calculated location, respectively (both with unit of mg/L); *t* and *x* are time (with unit of s) and calculated distance (with unit of m), respectively; *v* is seepage velocity (with unit of m/s); *R*_d_ is the retardation factor, and *D* is the coefficient of hydrodynamic dispersion (with unit of m^2^∙s). The *v* is a function of hydraulic conductivity *k*, hydraulic gradient *i*, and porosity *n* (Equation (7)); while the *R*_d_ is related to dry density *ρ*_d_ (with unit of g/cm^3^), porosity *n*, and partition coefficient *K*_p_ (Equation (8)).

(7) $$v=\frac{k\cdot i}{n}$$

(8) $$R_{d}=1+\frac{\rho_{d}}{n}K_{p}$$

Specifically, the *D* can be calculated by the following equation:

*D* = *τD*_0_ + *α*_L_*v* (9)

where *τ* is tortuosity factor; *D*_0_ is free-solution diffusion coefficient (m^2^·s); *α*_L_ is longitudinal dispersivity (m); and *v* is seepage velocity (m/s).

## 3. Results and Discussion

### 3.1. Hydraulic Conductivity Results

**Figure 2:**
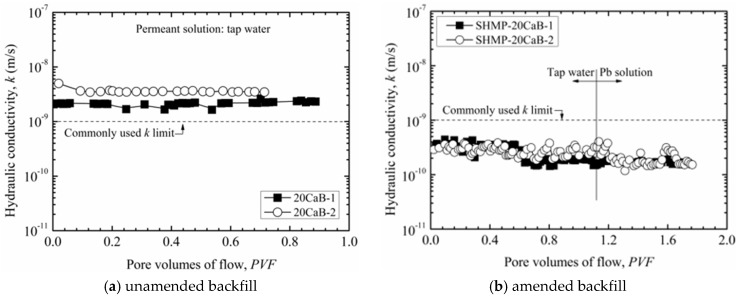
Hydraulic conductivities of the (**a**) unamended backfill (20CaB), and (**b**) amended backfill (SHMP-20CaB).

Figure 2 illustrates hydraulic conductivity as a function of pore volumes of flow for both unamended backfill (20CaB) and amended backfill (SHMP-20CaB), with the suffix ‘-*x*’ denoting the number of replicate sample. The average of the last four hydraulic conductivity values was used as the final hydraulic conductivity for each specimen. Without the SHMP amendment, the two tested 20CaB specimens exhibited hydraulic conductivity of 2.3 × 10^−9^ m/s and 3.5 × 10^−9^ m/s in tap water. These values are higher than the commonly recommended hydraulic conductivity of 10^−9^ m/s for SB slurry walls, thus the 20CaB backfill is considered unsuitable to use in SB wall construction. As the hydraulic conductivity was higher with tap water, no further testing was conducted with Pb solution as permeant liquid.

In contrast, the replicate SHMP-amended backfill specimens (SHMP-20CaB) exhibited hydraulic conductivity of 1.9 × 10^−10^ m/s and 3.0 × 10^−10^ m/s when tap water (*k*_w_) was used as permeant, and 1.7 × 10^−10^ m/s and 1.6 × 10^−10^ m/s when Pb solution (*k*_c_) was used as permeant. These results showed that Pb solution decreases hydraulic conductivity 0.89 to 0.53 times as compared to that with tap water as permeant. This result is contrary to the general concept of an increase in hydraulic conductivity of the SB backfill when an inorganic chemical solution is permeated, due to compression of diffused double layer of the bentonite. The decrease in hydraulic conductivity using Pb solution as permeant is attributed to the following reasons [18]: (1) the compression of diffused double layer thickness of the bentonite due to Pb^2+^ cation may be offset by the effective stress applied on the specimen during the testing period; and (2) precipitates formed due to complexation reaction between the SHMP and Pb^2+^ may have partially blocked the flow channels.

For evaluating chemical compatibility of the soils based on flexible wall hydraulic conductivity testing as per ASTM D7100, the concentration of the target contaminant concentration in the effluent should be within 0.9 to 1.1 times of that of the influent solution concentration [36]. However, the effluent Pb concentration measured in the tests conducted in this study was 4 to 5 orders of magnitude lower than the Pb concentration in the influent, which indicates chemical equilibrium has not been established within the specimens. Therefore, hydraulic conductivity with Pb solution, *k*_c_, obtained from this study only represents the short-term hydraulic conductivity of the tested specimen, and longer testing time is required to evaluate its long-term hydraulic conductivity variation (full chemical compatibility).

In the case of unamended backfill (20CaB), hydraulic conductivity value based on testing with tap water is used for Pb transport assessment. It should be noted that calcium bentonite generally possesses better chemical (e.g., heavy metals) compatibility than sodium bentonite [37]; therefore, it is reasonable to consider that the hydraulic conductivity of the 20CaB backfill remains unchanged even when permeating by chemical solution as compared to that of backfills containing sodium bentonite permeated with the same chemical solution. When SB backfill with 7.1 wt% sodium bentonite permeated firstly by tap water and then by 5 mM sodium chloride (CaCl_2_) solution, Bohnhoff and Shackelford [6] observed a *k*_c_-to-*k*_w_ ratio of 0.96. The 1000 mg/L (i.e., 4.8 mM) Pb solution tested in this study possesses essentially similar molar concentration as the 5 mM CaCl_2_ solution tested by Bohnhoff and Shackelford [6]. Thus, it is reasonable to use *k*_w_ instead of *k*_c_ for the Pb transport through 20CaB backfill in this study. A summary of final parameters of each backfill specimen is presented in Table 1.

### 3.2. Sorption Isotherm Results

**Figure 3:**
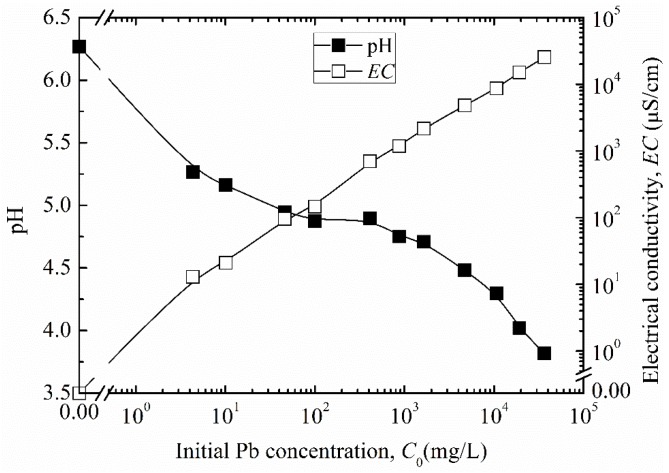
pH and electrical conductivity (*EC*) of the Pb solutions at different initial concentration.

Figure 3 presents the pH and *EC* results of the Pb solution as a function of initial concentration *C*_0_.

The pH values exhibit a decrease trend with Pb concentration increased, whilst the *EC* values show an increase trend with Pb concentration increased. This is attributed to acid property of the lead nitrate, and increased ionic concentration when more lead nitrate was dissolved into the deionized water.

**Figure 4:**
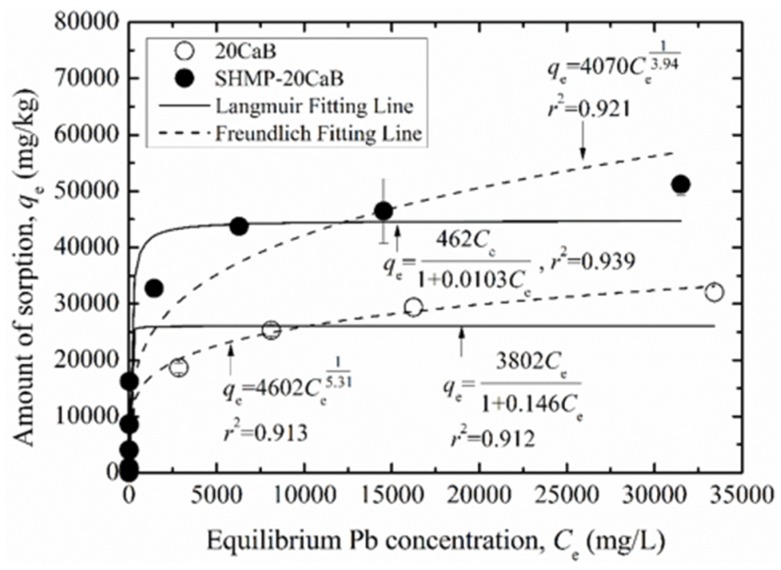
Langmuir and Freundlich sorption parameters for unamended and amended backfills.

Figure 4 shows the variation of the amount of sorption (*q*_e_) with equilibrium Pb concentration (*C*_e_) for unamended backfill (20CaB) as well as amended backfill (SHMP-20CaB). The Langmuir model and the Freundlich model are employed to describe the *q*_e_-*C*_e_ relationship of the testing backfills, and the fitting results are also included in Figure 4. It can be seen from Figure 4 that *q*_e_-*C*_e_ shows remarkable nonlinearity regardless of the types of backfill. There is a first sharp and then gradual increasing trend in the *q*_e_ as *C*_e_ value increases. The *q*_e_-*C*_e_ curve of the SHMP-20CaB is found above that of its unamended counterpart, i.e., 20CaB. It is worth noting that although only 4 visible data points are presented in gradual increasing portion of the *q*_e_-*C*_e_ curve for each backfill, there are additional 8 data points contained in the range of *C*_e_ values lower than 30 mg/L. The measured data are well fitted by the Langmuir model and Freundlich model with high coefficient of determination (*r*^2^) values. The values of fitted sorption model parameters and the calculated partition coefficient *K*_p_ values are summarized in Table 2.

Based on the Langmuir model, sorption capacities of the unamended and amended backfills are 26,044 mg/kg and 44,824 mg/kg, respectively. This indicates an increase in sorption capacity of the backfill by factor of 1.72 times with the SHMP amendment. The increase in Pb sorption capacity of the amended backfill is attributed to: (1) increased surface area of the CaB by the SHMP. The grain size of the CaB decreases and the surface area increases after amended by the SHMP [38], thus providing more sites for Pb sorption. As the CaB possesses lower surface area, the unamended backfill possesses lower sorption capacity; (2) increased surface charge density of the CaB by SHMP. The SHMP-CaB exhibits more negative zeta potential as compared to the CaB under the same testing solution [38], resulting in increased negative surface charge density on the amended bentonite particles and thus is conducive to the sorption of Pb; and (3) complexation between the SHMP and Pb may also be responsible for increased sorption capacity of the SHMP-CaB. The sorption intensity (*n*_F_) values of all the tested backfills (ranged from 3.94 to 5.31) is higher than 1.0, implying a favorable sorption.

### 3.3. Solute Transport Modeling

A summary of parameters for transport modeling is presented in Table 3. The *R*_d_ values in Table 3 are calculated using Equation 8 with *n* and *ρ*_d_ values shown in Table 1 and *K*_p_ values shown in Table 2. A tortuosity factor *τ* of 0.18 is adopted, and a *D*_0_ is obtained from Shackelford and Daniel [39], and found to be 9.25 × 10^−10^ m^2^·s for Pb cation when dispersing in infinite diluted solution. The value of longitudinal dispersivity *α*_L_ of 0.01 m as recommended by Kamon et al. [40] and Neville and Andrews [41] for soil-bentonite backfill is used for Pb transport analysis. The *D* is calculated according to Equation 9, in which the values of seepage velocity (*v*) are presented in Table 1.

**Figure 5:**
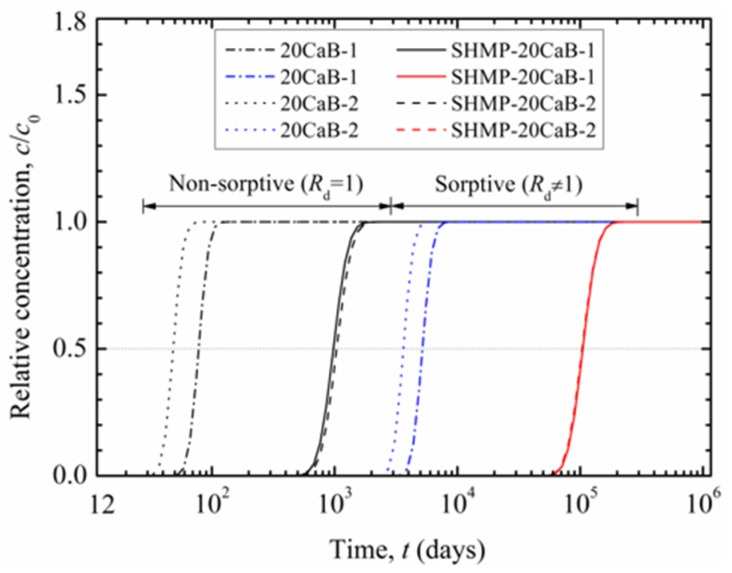
Predicted breakthrough curves for unamended and amended backfills with source Pb concentration = 1000 mg/L and wall thickness = 1 m.

Figure 5 shows breakthrough curves of 1000 mg/L Pb in a 1-m-thick SB slurry wall comprising with unamended backfill (20CaB) or amended backfill (SHMP-20CaB). The breakthrough curves for the no sorption scenario (*R*_d_ = 1), in which Pb sorption onto bentonite is assumed to be zero, is also shown in Figure 5 for comparison purpose. The replicate specimens exhibit similar breakthrough curves for both unamended and amended cases. Figure 5 indicates one to two orders of magnitude delay in the first arrival of the Pb front at the target boundary for the sorption case compared with that of no sorption case.

The time corresponding to relative concentration of 0.5 is commonly employed as the breakthrough time (*t*_B_) of target contaminant in many previous studies [42,43], while others also defined the time at relative concentration of 0.1 [44], 0.05 [45] or 0.01 [46] as *t*_B_. Depending on the source concentration, the contaminant concentration in the effluent (after transported through the slurry wall) may be higher than acceptable limit for groundwater, despite the relative concentration value is low. Therefore, *t*_B_ value at relative concentration of 0.05 to 0.5 may not be appropriate to use, especially when the source concentration is extremely high. To further understand the effects of variation in the *t*_B_ definition on the service life assessment of the slurry walls, Pb concentration limits in groundwater of Category III (i.e., Pb concentration no more than 0.01 mg/L) and Category IV (i.e., Pb concentration no more than 0.1 mg/L) as per “Groundwater Quality Standards” GB/T 14848-2017 [47] are assessed in this study to generate relative concentrations of 0.00001 and 0.0001, respectively. As a result, abbreviation *t*_B,*i*_ is used to represent breakthrough time defined with different relative concentration. For example, *t*_B,III_ and *t*_B,0.5_ mean breakthrough times for the effluent Pb concentration to be Category III groundwater standard (0.01 mg/L) and relative concentration of 0.5, respectively.

Predicted breakthrough times corresponding to relative concentration of 0.00001 (i.e., *t*_B,III_) to 0.5 (i.e., *t*_B,0.5_) for 1000 mg/L source Pb migrates through 1-m-thick wall are summarized in Table 4. The average *t*_B,*i*_ for the sorptive backfill is approximately two orders of magnitude of the average *t*_B,*i*_ for the (non-sorptive backfill. This increasing trend in *t*_B,*i*_ is consistent with that found in first arrival time shown in Figure 5.

The effects of SHMP amendment are also evident, *t*_B,*i*_ is increased by a factor of 12 to 14 (see *t*_B,amended/unamended_ in Table 4) with the amended backfill as compared to the unamended backfill under assumed non-sorptive condition. In contrast, when sorption is considered, *t*_B,*i*_ is increased from 18 to 24 times for amended backfill as compared to the unamended backfill. This is attributed to approximately 1.5 times increase in retardation factor *R*_d_ (see Table 3) combined with about 17 times decrease in seepage velocity *v* (see Table 1) due to SHMP amendment in the backfill. Overall, for test condition employed in this study, the transport of Pb through the 1-m-thcik cutoff wall will be significantly retarded with the amended backfill.

**Figure 6:**
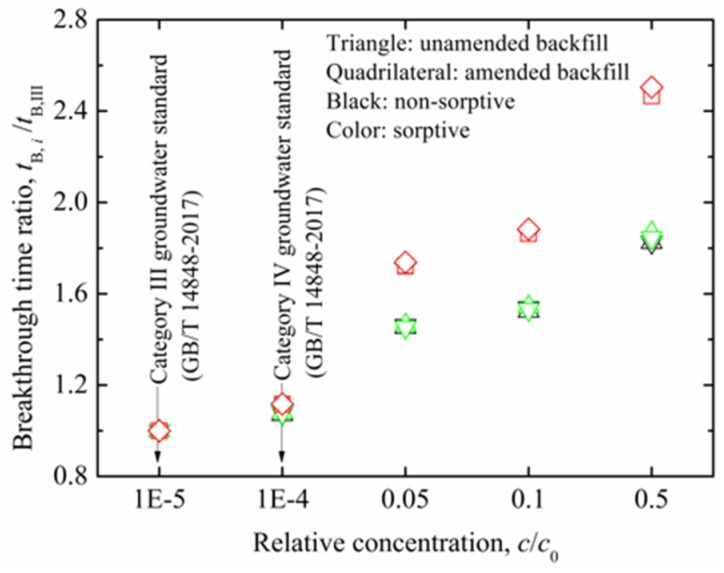
Relationship between breakthrough time ratio and relative concentration for unamended and amended backfills with source Pb concentration = 1000 mg/L and wall thickness = 1 m.

Figure 6 shows relationship between *t*_B,*i*_/*t*_B,III_ and relative concentration as a function of sorptive type of the backfills. The *t*_B,*i*_/*t*_B,III_ values for the non-sorptive amended backfill is observed to overlap with those of sorptive amended backfill under the same relative concentration standard. The same trend can be observed for the unamended backfill scenario. This finding is attributed to the parallel breakthrough curves of the non-sorptive amended backfill (or non-sorptive unamended backfill) and the sorptive amended backfill (or sorptive unamended backfill). In contrast, *t*_B,*i*_/*t*_B,III_ for all of the backfills exhibits increasing trend with increasing relative concentration, with increase in *t*_B,*i*_/*t*_B,III_ of the amended backfill more significant than that in *t*_B,*i*_/*t*_B,III_ of the unamended backfill. For example, *t*_B,*i*_/*t*_B,III_ value increases from 1 to 1.9 and 1 to 2.5 in unamended backfill and amended backfill, respectively, when relative concentration increases from 0.00001 (*c*/*c*_0_ at Category III groundwater standard) to 0.5. These results indicate that the relative concentration used for defining breakthrough time can have remarkable influence on breakthrough times of the slurry wall, with higher relative concentration values resulting lower breakthrough times. The *t*_III_ and *t*_IV_ yield a conservative result, i.e., shorter breakthrough time, as compared to the *t*_0.05_, *t*_0.1_, and *t*_0.5_. Therefore, the impacts of source contaminant concentration together with its groundwater quality standard should be considered when evaluating longevity and containment performance of the SB slurry walls.

## 4. Study Limitations

Only one type of heavy metal Pb was used in this study for investigating hydraulic conductivity, sorption, and containment performance of the SB backfill, but multiple heavy metals may co-exist in groundwater and the effects of these multiple contaminants should be evaluated in future studies. An effective stress of 34.5 kPa is employed in laboratory hydraulic conductivity tests conducted in this study, which may be higher than that expected under field conditions [48], hence the effect of confining pressure needs further evaluation. The hydraulic conductivities testing with Pb solution were obtained without achieving chemical equilibrium, therefore, the result only represents the short-term hydraulic conductivity, and longer testing time is needed to access the long-term hydraulic conductivity of the sand/SHMP-amended bentonite backfill. In addition, the amended backfill and the unamended backfill are assumed to possess the same tortuosity and dispersivity, which may also need further examination.

## 5. Conclusions

This study investigated the effects of amending calcium-bentonite (CaB) with 2 wt% (dry weight) of sodium hexametaphosphate (SHMP) on hydraulic conductivity and Pb sorption/retardation in soil/CaB slurry trench cutoff wall. Hydraulic conductivity was determined with flexible-wall permeability tests, sorption capacity was characterized via batch sorption experiences, and containment performance was assessed by the transport modeling through a 1-m-thick slurry wall. Based on this study, the following conclusions are drawn:

The SHMP amendment decreased hydraulic conductivity of the soil/CaB backfill containing 20 wt% CaB by an order of magnitude when permeated with tap water. The amended backfill exhibited excellent compatibility to 1000 mg/L Pb solution during the tested period (short-term), with insignificant variation observed in the hydraulic conductivity with time as permeated liquid changed from tap water (i.e., 1.9 to 3.0 × 10^−10^ m/s) to Pb solution (1.6 to 1.7 × 10^−10^ m/s).

Both unamended and amended backfills displayed nonlinear Pb sorption behavior that could be described well by the Langmuir or Freundlich model. The SHMP amendment increased Pb sorption capacity of the soil/CaB backfill by a factor of 1.72, yielding 1.5 times increase in retardation factor of the amended backfill relative to the unamended one.

The SHMP had potential to retard Pb breakthrough time through a 1-m-thick slurry wall by two orders of magnitude as compared to its non-sorptive backfill counterpart. Breakthrough time at relative concentration of 0.00001 (Category III groundwater standard) to 0.5 for the amended backfill was 12 to 24 times of that for the unamended backfill, which was attributed to increased retardation factor combining with decreased seepage velocity of the backfill after SHMP amendment.

Ratio of breakthrough time at various relative concentrations to that at Category III groundwater standard *t*_B,*i*_/*t*_B,III_ increased with increasing relative concentration selected for defining the breakthrough time. The source concentration level and groundwater safety/quality standard should be seriously considered when evaluating service life and containment performance of the SB slurry walls. Overall, the findings of this study demonstrate that SHMP may be considered as an effective amendment for improving Pb containment performance of a soil/CaB slurry wall.

## Figures and Tables

**Table 1:** Summary of final parameters of specimen after flexible wall hydraulic conductivity tests.

Sample ID	Hydraulic Conductivity, *k* (m/s)	Hydraulic Gradient, *i*	Porosity, *n*	Seepage Velocity, *v* (m/s)	Specimen Height, *L* (m)	Dry Density, *ρ*_d_ (g/cm^3^)
20CaB-1	2.3 × 10^−9^	26	0.413	1.47 × 10^−7^	0.0785	1.60
20CaB-2	3.5 × 10^−9^	26	0.384	2.37 × 10^−7^	0.0693	1.68
SHMP-20CaB-1	1.7 × 10^−10^	26	0.371	1.16 × 10^−8^	0.0733	1.68
SHMP-20CaB-2	1.6 × 10^−10^	26	0.390	1.09 × 10^−8^	0.0753	1.64

**Table 2:** Summary of best-fit Langmuir and Freundlich parameters for Pb sorption in backfill mixtures.

**Type of Backfill**	**Langmuir Parameters**			
	***q*_m,L_ (mg/kg)**	***K*_L_ (L/mg)**		***r*^2^**
20CaB	26,044	0.146		0.912
SHMP-20CaB	44,824	0.0103		0.939
	**Freundlich Parameters**			***K*_p_ at** ***C*_e_ = 1000 mg/L**
	***K_F_* (L/kg)**	***n*_F_**	***r*^2^**	
20CaB	4602	5.31	0.913	16.9
SHMP-20CaB	4070	3.94	0.921	23.5

**Table 3:** Summary of transport parameters for Pb in backfills.

Type of Backfill	*R*_d_	*τ*	*D*_0_ (m^2^∙s)	*α*_L_ (m)	*D* (m^2^∙s)
20CaB-1	66.47	0.18	9.25 × 10^−10^	0.01	1.64 × 10^−9^
20CaB-2	74.94	0.18	9.25 × 10^−10^	0.01	2.54 × 10^−9^
SHMP-20CaB-1	107.42	0.18	9.25 × 10^−10^	0.01	2.83 × 10^−10^
SHMP-20CaB-2	99.82	0.18	9.25 × 10^−10^	0.01	2.76 × 10^−10^

**Table 4:** Summary of predicted breakthrough times for 1000 mg/L Pb transport through a 1-m-thick SB slurry wall.

Breakthrough Time, *t*_B,*i*_ (yrs)	Unamended Backfill			Amended Backfill			*t*_B_ Ratio ^a^, *t*_B,amended/unamended_
	20CaB-1	20CaB-2	Average	SHMP-20CaB-1	SHMP-20CaB-2	Average	
**Non-sorptive**							
*t*_B,III_	0.12	0.07	0.09	1.08	1.13	1.11	12
*t*_B,IV_	0.12	0.08	0.10	1.21	1.27	1.24	12
*t*_B,0.05_	0.17	0.10	0.14	1.86	1.97	1.92	14
*t*_B,0.1_	0.18	0.11	0.14	2.02	2.13	2.08	15
*t*_B,0.5_	0.21	0.13	0.17	2.67	2.84	2.76	16
**Sorptive**							
*t*_B,III_	7.59	5.38	6.48	116.24	113.08	114.66	18
*t*_B,IV_	8.21	5.80	7.01	129.66	126.35	128.00	18
*t*_B,0.05_	11.11	7.81	9.46	200.26	196.58	198.42	21
*t*_B,0.1_	11.72	8.23	9.98	216.63	212.94	214.79	22
*t*_B,0.5_	14.18	9.92	12.05	286.68	283.25	284.97	24

^a^ The *t*_B_ ratio is calculated based on the “average” values of corresponding unamended and amended backfills.

## References

[1] Sharma H.D., Reddy K.R. (2004). Geoenvironmental Engineering: Site Remediation, Waste Containment, and Emerging Waste Management Technologies.

[2] Shackelford C.D., Jefferis S.A. Geoenvironmental engineering for in situ remediation. Proceedings of the International Conference on Geotechnical and Geoenvironmental Engineering.

[3] Li Y.C., Cleall P.J., Wen Y.D., Chen Y.M., Pan Q. (2015). Stresses in soil-bentonite slurry trench cutoff walls. Géotechnique.

[4] Zhang W.J., Chen G.U., Lou X.H. (2017). Measurement of hydraulic conductivity and diffusion coefficient of backfill for soil-bentonite cutoff wall under low consolidation pressure. Chin. J. Geotech. Eng..

[5] Malusis M.A., McKeehan M.D. (2013). Chemical compatibility of model soil-bentonite backfill containing multiswellable bentonite. J. Geotech. Geoenviron. Eng..

[6] Bohnhoff G.L., Shackelford C.D. (2014). Hydraulic conductivity of polymerized bentonite-amended backfills. J. Geotech. Geoenviron. Eng..

[7] Du Y.J., Fan R.D., Liu S.Y., Reddy K.R., Jin F. (2015). Workability, compressibility and hydraulic conductivity of zeolite-amended clayey soil/calcium-bentonite backfills for slurry-trench cutoff walls. Eng. Geol..

[8] Hong C.S., Shackelford C.D., Malusis M.A. (2016). Adsorptive behavior of zeolite-amended backfills for enhanced metals containment. J. Geotech. Geoenviron. Eng..

[9] Malusis M.A., Maneval J.E., Barben E.J., Shackelford C.D., Daniels E.R. (2010). Influence of adsorption on phenol transport through soil–bentonite vertical barriers amended with activated carbon. J. Contam. Hydrol..

[10] Khandelwal A., Rabideau A.J. (2000). Enhancement of soil-bentonite barrier performance with the addition of natural humus. J. Contam. Hydrol..

[11] Du Y.J., Fan R.D., Liu S.Y., Reddy K.R., Jin F. (2015). Impacts of presence of lead contamination in clayey soil–calcium bentonite cutoff wall backfills. Appl. Clay Sci..

[12] Du Y.J., Yang Y.L., Fan R.D., Wang F. (2016). Effects of phosphate dispersants on the liquid limit, sediment volume and apparent viscosity of clayey soil/calcium-bentonite slurry wall backfills. KSCE J. Civ. Eng..

[13] Fan R.D., Liu S.Y., Du Y.J., Reddy K.R., Yang Y.L. (2017). Impacts of presence of lead contamination on settling behavior and microstructure of clayey soil-calcium bentonite blends. Appl. Clay Sci..

[14] Adebowale K.O., Unuabonah I.E., Olu-owolabi B.I. (2006). The effect of some operating variables on the adsorption of lead and cadmium ions on kaolinite clay. J. Hazard. Mater..

[15] Ma M. (2012). The dispersive effect of sodium hexametaphosphate on kaolinite in saline water. Clays Clay Miner..

[16] Deng A., McBride L. Hydraulic conductivity of Hindmarsh clay amended by polymeric additive. Proceedings of the 7th International Congress on Environmental Geotechnics (ICEG 2014).

[17] Yang Y.L., Du Y.J., Reddy K.R., Fan R.D. (2017). Phosphate-amended sand/Ca-bentonite mixtures as slurry trench wall backfills: Assessment of workability, compressibility and hydraulic conductivity. Appl. Clay Sci..

[18] Yang Y.L., Reddy K.R., Du Y.J., Fan R.D. (2018). Short-term hydraulic conductivity and consolidation properties of soil-bentonite backfills exposed to CCR-impacted groundwater. J. Geotech. Geoenviron. Eng..

[19] Yang Z.P., Lu W.X., Long Y.Q., Bao X.H., Yang Q.C. (2011). Assessment of heavy metals contamination in urban topsoil from Changchun City, China. J. Geochem. Explor..

[20] Yang Z.P., Ge H.K., Lu W.X., Long Y.Q. (2015). Assessment of heavy metals contamination in near-surface dust. Pol. J. Environ. Stud..

[21] Xia W.Y., Du Y.J., Li F.S., Li C.P., Yan X.L., Arulrajah A., Wang F., Song D.J. (2019). In-situ solidification/stabilization of heavy metals contaminated site soil using a dry jet mixing method and new hydroxyapatite based binder. J. Hazard. Mater..

[22] Xia W.Y., Du Y.J., Li F.S., Guo G.L., Yan X.L., Li C.P., Arulrajah A., Wang F., Wang S. (2019). Field evaluation of a new hydroxyapatite based binder for ex-situ solidification/stabilization of a heavy metal contaminated site soil around a Pb-Zn smelter. Constr. Build. Mater..

[23] Yang Y.L., Reddy K.R., Du Y.J., Fan R.D. (2019). Retention of Pb and Cr (VI) onto slurry trench vertical cutoff wall backfill containing phosphate dispersant amended Ca-bentonite. Appl. Clay Sci..

[24] ASTM (2012). Standard Test Methods for Slump of Hydraulic-Cement Concrete.

[25] Evans J.C., Daniel D.E. (1993). Vertical cutoff walls. Geotechnical Practice for Waste Disposal.

[26] ASTM (2010). Standard Test Methods for Measurement of Hydraulic Conductivity of Saturated Porous Materials Using a Flexible Wall Permeameter.

[27] Malusis M.A., Barben E.J., Evans J.C. (2009). Hydraulic conductivity and compressibility of soil-bentonite backfill amended with activated carbon. J. Geotech. Geoenviron. Eng..

[28] ASTM (2015). Standard Test Method for pH of Aqueous Solutions with the Glass Electrode.

[29] ASTM (2014). Standard Test Method for Electrical Conductivity and Resistivity of Water.

[30] ASTM (2008). Standard Test Method for 24-h Batch-Type Measurement of Contaminant Sorption by Soils and Sediments.

[31] Reddy K.R., Xie T., Dastgheibi S. (2014). Removal of heavy metals from urban stormwater runoff using different filter materials. J. Environ. Chem. Eng..

[32] US EPA (2007). SW-846 Test Method 7000B: Flame Atomic Absorption Spectrophotometry.

[33] Langmuir I. (1916). The constitution and fundamental properties of solids and liquids. Part I. Solids. J. Am. Chem. Soc..

[34] Freundlich H.M.F. (1906). Over the adsorption in solution. J. Phys. Chem..

[35] Shackelford C.D., Daniel D.E. (1993). Contaminant transport. Geotechnical Practice for Waste Disposal.

[36] ASTM (2011). Standard Test Methods for Hydraulic Conductivity Compatibility Testing of Soils with Aqueous Solutions.

[37] Gleason M.H., Daniel D.E., Eykholt R. (1997). Calcium and sodium bentonite for hydraulic containment applications. J. Geotech. Geoenviron. Eng..

[38] Yang Y.L., Reddy K.R., Du Y.J., Fan R.D. (2018). SHMP amended calcium bentonite for slurry trench cutoff walls: Workability and microstructure characteristics. Can. Geotech. J..

[39] Shackelford C.D., Daniel D.E. (1991). Diffusion in saturated soil. I: Background. J. Geotech. Eng..

[40] Kamon M., Inui T., Katsumi T. Environmental risk assessment of a containment disposal facility at a contaminated site. Proceedings of the 12th Asian Regional Conference on Soil Mechanics and Geotechnical Engineering.

[41] Neville C.J., Andrews C.B. (2006). Containment criterion for contaminant isolation by cutoff walls. Ground Water.

[42] Shackelford C.D. (1994). Critical concepts for column testing. J. Geotech. Eng..

[43] Li L.Y., Li F. (2001). Heavy metal sorption and hydraulic conductivity studies using three types of bentonite admixes. J. Environ. Eng..

[44] Zhang W.J., Qiu Q.W. (2010). Analysis on contaminant migration through vertical barrier walls in a landfill in China. Environ. Earth Sci..

[45] Hong C.S., Shackelford C.D., Honoring David E., Daniel Benson C.H., Shackelford C.D. (2016). Characterizing zeolite-amended soil-bentonite backfill for enhanced metals containment with vertical cutoff walls. Geoenvironmental Engineering.

[46] Wang Y.Z., Chen Y.M., Xie H.J., Zhang C., Zhan L. (2016). Lead adsorption and transport in loess-amended soil-bentonite cut-off wall. Eng. Geol..

[47] AQSIQ (General Administration of Quality Supervision, Inspection and Quarantine of the People’s Republic of China), SAC (Standardization Administration of the People’s Republic of China) (2017). Groundwater Quality Standards.

[48] Evans J.C., Yang Y.L., Ruffing D.G. Vane shear tests to evaluate in situ stress state of a soil-bentonite slurry trench wall. Proceedings of the International Congress on Environmental Geotechnics.

